# Commentary: Association between Life's Crucial 9 and overactive bladder: the mediating role of weight-adjusted-waist index

**DOI:** 10.3389/fnut.2025.1651989

**Published:** 2025-08-25

**Authors:** Rui Du, Yuzhe Su, Peng Zhang

**Affiliations:** ^1^The First Affiliated Hospital of Dalian Medical University, Dalian, China; ^2^Department of General Surgery, The First Affiliated Hospital of Dalian Medical University, Dalian, China; ^3^Department of Urology, The First Affiliated Hospital of Dalian Medical University, Dalian, China

**Keywords:** Life's Crucial 9 (LC9), overactive bladder, weight-adjusted-waist index (WWI), National Health and Nutrition Examination Survey (NHANES), mediation analysis

## 1 Introduction

We read with interest the article by Gong et al. entitled “Association between Life's Crucial 9 and overactive bladder: the mediating role of weight-adjusted-waist index” (1). This study is the first to explore the association between Life's Crucial 9 (LC9) and overactive bladder (OAB) using data from National Health and Nutrition Examination Survey (NHANES) 2005–2018. Through multivariable logistic regression models, the authors found that each 10-point increase in LC9 was associated with a 28% lower risk of OAB [odds ratio (OR): 0.72; 95% confidence interval (CI): 0.69–0.76]. Restricted cubic spline (RCS) analysis demonstrated a significant linear negative correlation between LC9 and OAB. The subgroup analyses validated the robustness of the LC9-OAB relationship across diverse populations. Additionally, mediation analysis showed that 13.89% of the relationship between LC9 and OAB was mediated by the weight-adjusted waist index (WWI). This novel indicator demonstrates superior ability in distinguishing fat from muscle mass, thereby providing a more accurate assessment of visceral adiposity compared to traditional obesity metrics. The study by Huang et al. further supports WWI's enhanced predictive capability for OAB relative to other anthropometric indices (2). Collectively, this study provides new epidemiological evidence for the prevention of OAB through improvements in cardiovascular health and obesity management. However, we have deeper reflections on this study.

## 2 Predictive performance

In 2010, the American Heart Association (AHA) introduced Life's Simple 7 (LS7) to assess cardiovascular health (3). In 2022, Life's Essential 8 (LE8) added sleep health (4). In 2024, mental health was incorporated into the updated LC9 (5). The LC9 score ultimately includes nine elements: diet, physical activity, nicotine exposure, sleep health, BMI, lipids, blood glucose, blood pressure, and mental health (1). The evolution of cardiovascular health assessments, from the original LS7 to updated LE8 and current LC9, highlights an increasing recognition of the multifaceted nature of health. However, the integration of additional components such as sleep and mental health has raised important questions regarding the practicality and predictive efficacy of these expanded models. A previous study on mortality prediction demonstrated that neither LE8 nor LC9 showed superior predictive performance compared to LS7 (6). Moreover, the complexity introduced by adding sleep and mental health may detract from the usability of LC9 in clinical settings, where straightforward and actionable metrics are often preferred. Therefore, we recommend using receiver operating characteristic (ROC) curve to evaluate the predictive performance of LS7, LE8 and LC9 for OAB. If the findings are similar to the previous study, then for practical use in clinical and public health, simplifying this cardiovascular health score could be considered.

## 3 Covariate adjustment

In this study, we noted that the authors accounted for all covariates in model 3, including all demographic variables as well as smoking, alcohol consumption, hypertension, diabetes, and dyslipidemia (1). According to the AHA's definition of LC9, some of the covariates adjusted for in model 3 are highly correlated with the components of LC9 (1). The authors didn't mention if they did a collinearity analysis; as a result, we think that the adjustments for smoking, hypertension, diabetes, and dyslipidemia in model 3 might have changed the study's conclusions: the real effect of LC9 might have been diluted, and the final OR value may underestimate the true strength of the association. We recommend supplementing the calculation of variance inflation factors (VIF) to assess the degree of multicollinearity among variables. Variables with VIF >5 are considered to exhibit high multicollinearity and should be excluded (7).

## 4 Discussion

In conclusion, this study presents an innovative investigation into the association between LC9 and OAB in the American population. However, we hope the authors will consider our suggestions to further enrich the understanding of the LC9-OAB relationship and provide additional insights into OAB in the future.
